# Impact of Muscle Changes Assessed by Ultrasonography on Muscle Strength and Functioning after ICU Discharge: A Systematic Review with Meta-Analysis

**DOI:** 10.3390/ijerph21070908

**Published:** 2024-07-11

**Authors:** Felipe Douglas Silva Barbosa, Brenda Stephanie Santos Nascimento, Maysa Carolina de França Souza Silva, Telma Cristina Fontes Cerqueira, Valter Joviniano de Santana Filho

**Affiliations:** 1 Department of Family Health and Occupational Therapy, Faculty of Medicine, Federal University of Bahia, Salvador 40026-010, BA, Brazil; 2 Post-Graduate Program in Health Sciences, Federal University of Sergipe, Aracaju 49060-100, SE, Brazil; vjsf@academico.ufs.br; 3 Department of Physioterapy, Campus Lagarto, Federal University of Sergipe, Lagarto 49400-000, SE, Brazil; brendasantosn1@gmail.com (B.S.S.N.); fisioterapiamaysa@gmail.com (M.C.d.F.S.S.); telmacristina@academico.ufs.br (T.C.F.C.); 4 Department of Physioterapy, Campus São Cristóvão, Federal University of Sergipe, São Cristóvão 49100-000, SE, Brazil

**Keywords:** ultrasonography, muscle assessment, strength, mobility, critical care

## Abstract

Background: Ultrasonography has been used to identify structural, quantitative, and qualitative muscle changes. These changes have been assessed in different muscles during ICU stays; however, it is unclear if it can predict functioning after ICU discharge. Objective: To analyze the relationship between muscle changes assessed by ultrasonography and the strength and functioning of ICU survivors. Methods: A systematic review with a meta-analysis was performed according to the MOOSE guidelines and registered in PROSPERO. Searches of the following databases were performed by two of the authors: PubMed, Cinahl, Embase, Scopus, LILACS, Web of Science, and Science Direct. Qualitative analysis was performed using NOS and AHRQ scales. Meta-analysis was performed using the “R”, “metafor” package. Heterogeneity was assessed by I2 and Cochran’s Q test. Meta-regression analyses were performed to verify the moderators, and funnel plots and Egger’s regression intercept test were used to analyze the publication bias. Results: Sixteen articles were included in the qualitative assessment, and nine were used in the quantitative assessment. There is evidence of correlations between MT and muscle strength (r = 0.20 [0.11; 0.27]; *p* < 0.0001), and MT (r = 0.35 [0.19; 0.49]; *p* < 0.0001), CSA (r = 0.30 [0.10; 0.47]; *p* = 0.0038), EI (r = −0.29 [−0.53; −0.01]; *p* = 0.043) and mobility. In the subgroup analyses, some evidence of a correlation between specific muscles and strength and mobility were found. Conclusions: There is evidence for the correlation between muscle characteristics assessed by US and functioning outcomes.

## 1. Introduction

Individuals who have had critical illnesses, despite having a higher survival rate, experience serious long-term physical, cognitive, and mental impairments [1,2,3]. These impacts can lead to major changes in the ability to perform daily activities, social participation, economic impacts, and family dynamics, among others, in addition to a higher need for health services, chance of hospital readmission, and risk of mortality [3,4,5].

Several conditions can influence the occurrence of these impairments, including changes in muscle structure and function, which can lead to temporary or permanent difficulties in the functional capacity of critically ill patients [6,7]. Several factors can lead to muscle changes, including age, the criticality of the disease, immobility, previous functional status, and catabolic state, among others, which can result in a reduction in muscle mass and quality, in addition to intensive care unit-acquired weakness (ICUAW) [8].

Thus, ultrasonography (US) has been used for the quantitative and qualitative assessment of muscular changes in critically ill patients. Studies have analyzed the use of ultrasonography for muscle assessment since the early 2000s; however, there was a significant increase in the number of publications in 2012 and 2013 [9]. The initial studies sought to describe the principles of the neuromuscular US technique, with subsequent analyses of the ultrasound differences in the muscles of participants with a critical illness during hospitalization, the reliability of the assessment, and the relationship between the changes observed and the clinical and functional outcomes [9,10].

These analyses were performed based on the various characteristics of the peripheral skeletal muscle architecture, including muscle thickness (MT), cross-sectional area (CSA), and echointensity (EI) [11]. In addition, the fiber pennation angle and fascicle length have also been mentioned in muscle assessments, but they have not been widely used in the literature [10,11]. Thus, considering that a loss of muscle mass was already observed in critically ill patients admitted to intensive care units (ICUs), researchers have sought to analyze how these muscular changes occurred. In comparison to a group of healthy individuals, a significant decrease in biceps brachii, forearm flexors, and quadriceps femoris MT was observed in 16 patients at ICU discharge. However, the same was not observed in the diaphragm MT [12].

When analyzing the changes in different muscle characteristics assessed by US, studies have presented different methodologies and results. Gruther et al. [12] found that there was a significant reduction in quadriceps femoris MT during the first 28 days of hospitalization. Cartwright et al. [13], who evaluated the biceps brachii, abductor digiti minimi, rectus femoris, tibialis anterior, and diaphragm muscles, observed a significant worsening of muscle quality (EI) only in the quadriceps femoris and tibialis anterior after 14 days of hospitalization, but they did not observe this same result for the MT of the muscles evaluated. Puthucheary et al. [14] reported that there were significant reductions in the CSA of the rectus femoris on the 10th day of an ICU stay.

Taking into consideration the variety of methodologies and results in the literature, in addition to US assessment being evaluator-dependent—that is, it depends on the evaluator’s interpretation—inter- and intra-examiner reliability studies have been carried out. Reliability has also been analyzed considering the different types of ICUs, professional training, experience with US, and established protocols. Regarding the protocols analyzed, most studies evaluated one or two muscles or muscle groups, for one of the characteristics of their architecture.

The reliability of MT is the most widespread in the literature, and studies have demonstrated the excellent inter- and intra-examiner reliability of examiners, considering the previously mentioned variables, as well as for the CSA and EI for the assessment of the quadriceps femoris [15,16,17,18], tibialis anterior [15,16], biceps brachii [16,19,20], and diaphragm [18,21,22]. Furthermore, it has been demonstrated that changes in muscle mass and quality may be associated with changes in strength and functional capacity. It has been reported, in different ways, that decreases in MT and CSA and an increase in EI are mainly associated with reduced muscle strength and functional capacity, with less emphasis on the latter, in critically ill patients [6,23,24,25,26,27].

Despite all the recommendations for the use of US in muscle assessment, there are still disparities in the research regarding study methodology [28,29]. This fact has generated great diversity in the results found, which has led to difficulties in the comparability, verification, and generalization of results. Consequently, it is necessary to investigate the studies that have already been carried out, seeking to identify whether the disparities have influenced the results of the original studies, and to expand the representativeness and robustness of the scientific evidence for these outcomes. Therefore, this study aims to analyze the impact of muscle changes assessed by US on muscle strength and functional capacity after ICU discharge.

## 2. Materials and Methods

### 2.1. Study Type

This is a systematic review with meta-analysis in accordance with the Meta-analysis Of Observational Studies in Epidemiology (MOOSE) guidelines [30]. The protocol for this systematic review was developed and registered in the PROSPERO database with registration CDR42022361643. Thus, the research was carried out in four phases: identification, screening, eligibility, and inclusion. All stages were carried out between October 2022 and June 2023.

### 2.2. Eligibility Criteria

Eligibility criteria included observational studies (cross-sectional, case–control, cohort); studies in English, Portuguese, or Spanish; research with humans over 18 years old; studies that used US as an assessment method; studies that evaluated the relationship between muscle changes assessed by US and components of functioning; and studies without restrictions on race, sex, ethnicity, or comorbidities of research subjects. The exclusion criteria were laboratory studies, letters, summaries, comments, and case reports; duplicate studies; and studies carried out in pediatric populations.

### 2.3. Study Design

This systematic review established the guiding research question based on the population, exposure, comparator, and outcomes (PECO) strategy, with P—population, patient, or problem (individuals who survived critical illness), E—exposure (having muscular changes assessed using US), C—comparator (no muscular changes), O—outcome of interest (strength and functional capacity). Therefore, the following research question was created: “Is there an association between muscle changes assessed through ultrasound and the strength and functional capacity of critically ill patients after discharge from the ICU?”

The search terms were then established based on the guiding question, checking whether the terms were in the MESH classification. The fixed terms and entry terms were selected for subsequent searching in the databases.

In the first stage, searches were carried out in the following databases: CINAHL, EMBASE, LILACS, PubMed, Science Direct, Scopus, and Web of Science, starting in October 2022. Different combinations of the following keywords were used: “Muscle”; “Intensive care unit acquired weakness”; “Ultrasound”, “Ultrasonography”; “Intensive care unit”; “Critical care”, with Boolean operators AND and OR, if necessary. Full search strategies are available in Supplementary Materials S1.

In the second stage, researchers selected the articles found in the database by reading titles and abstracts. Subsequently, the articles were read in full and selected for inclusion in the study. Furthermore, seeking to make every effort to include articles in the review, various strategies were carried out, including sending direct e-mails to authors and manual searches in the references of selected articles.

Data were extracted from the articles to compose the result matrix, including title, authors, year of publication, main aim, number of participants, muscle characteristics assessed by US, assessments used to identify functioning outcomes, and outcome results of interest. All stages were conducted by three authors (two undergraduate physiotherapy students and a doctoral candidate) independently and blindly, with the third author acting to solve discrepancies during the process of identification, selection, inclusion, and evaluation of articles.

### 2.4. Quality Assessment

The quality of case–control and cohort studies was assessed using the Newcastle-Ottawa Quality Assessment Scale (NOS), while cross-sectional studies were assessed using the Agency for Healthcare Research and Quality scale (AHRQ scale). NOS evaluates the quality of studies based on 3 criteria: selection, comparability, and outcome. Studies with scores from 0 to 3 were considered “low”, 4 to 6 “moderate”, and 7 to 9 “high” quality. [31]. The AHRQ scale considers 11 items to assess quality, classifying articles with scores from 0 to 3 as “low”, 4 to 7 as “moderate” (4–7), and 8 to 11 as “high” quality [32].

### 2.5. Statistical Analysis

The meta-analysis was performed using the R environment for statistical computing and graphics, “metafor” package library (R Core Team version 4.2.1), using the random effects model. Effect sizes were converted to Pearson’s r when possible, if not presented in the included studies. The effect size r of the correlation between muscle changes assessed by US and the strength or functional capacity of patients after admission to the ICU in each study was used as primary data for the meta-analysis. As Pearson’s r is not normally distributed, these values were converted to Fisher’s z scale. Subgroup analyses were performed when possible to analyze the correlation of a muscle/muscle group with an assessment of the strength or functional capacity outcome, with at least three analyses of the original articles.

Heterogeneity of the studies was quantified by I^2^ measurements and evaluated using Cochran’s Q test. The I^2^ results were interpreted considering 0% as no heterogeneity between studies; less than 50% as low heterogeneity; between 50 and 75% as moderate heterogeneity; and greater than 75% as high heterogeneity [33]. Considering the possibility of heterogeneity between studies, meta-regression analysis was performed, including the variables “mean age of participants”, “time between the first and last US assessment”, and “methodological quality of studies” as moderators. Results were presented in forest plots, which show the effect sizes and confidence intervals (CIs) of the included studies, and the effect size and CIs of the analysis. Funnel plots and Egger’s regression intercept test were used to analyze publication bias. Sensitivity analyses were included to assess the robustness of the results, being performed for analyses that showed high heterogeneity between studies.

## 3. Results

### 3.1. Study Selection

**Figure 1:**
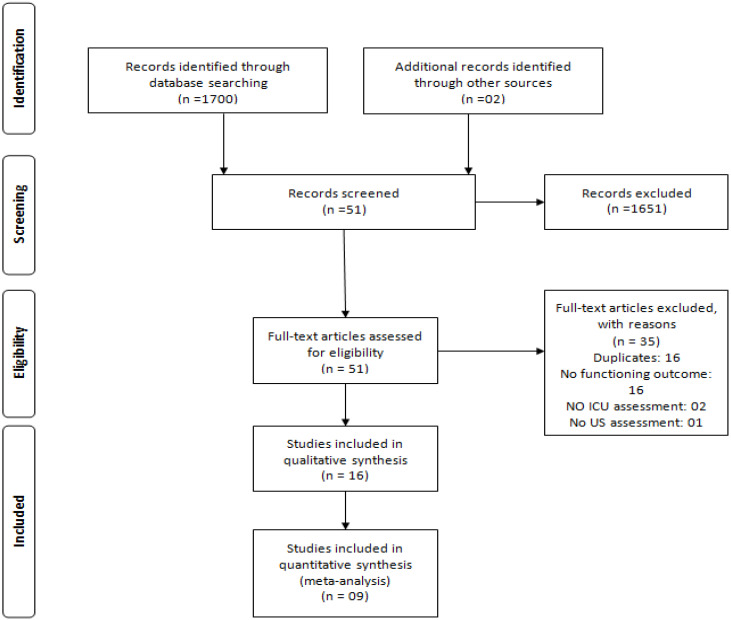
Study selection flowchart.

After applying the search strategies, 1700 articles were retrieved from the databases. After reading the titles and abstracts, 49 articles were selected for full-text screening, 35 of which were excluded after this stage. The reasons for exclusion were as follows. Sixteen were duplicates, sixteen did not address the relationship between the functioning outcome and the muscular changes assessed by US, two did not perform their assessment in the ICU, and one article did not use US as an assessment method. Additionally, two articles were retrieved through a manual search of included studies’ references, totaling 16 [6,7,23,24,34,35,36,37,38,39,40,41,42,43,44] articles included in this review. The detailed flowchart is shown in Figure 1. 

A summary of the data retrieved from the 16 studies included in this systematic review is presented in Table 1.

### 3.2. Type and Quality of Studies

Of the studies included, fifteen are prospective cohorts and one is a cross-sectional study. Of the cohort studies, ten were of high quality and five were of moderate quality. The cross-sectional study received a score of ten, considered high quality. Overall, 73.3% (11) of the included studies were of high quality and 26.7% (5) of moderate quality. Qualitative analyses are shown in Table 2 and Table 3.

### 3.3. Study Characteristics

Studies to verify the feasibility and reliability of muscle assessment using US in the ICU are recent, and the exploration of this assessment resource and the identification of the relationship with clinical and functional outcomes are even more contemporary, especially in the last ten years. The oldest article included in this study is from 2015, and fourteen of the included articles were published from 2018 onwards. Regarding the number of participants, most studies presented small, but representative samples, considering that the majority present a sample calculation. In this study were included 613 participants, with an incidence of 64.60% male. Considering all the studies analyzed, the average number of participants was 38.31 ± 21.29, with the study carried out by Witteveen et al. [39] presenting the largest number of participants (71 participants).

### 3.4. Muscle Characteristics Assessed by US

Of the sixteen studies included, thirteen evaluated MT [6,7,23,24,34,35,36,38,39,40,41,44,45], ten the CSA [6,7,19,23,24,34,36,40,42,45], eight the EI [6,7,23,35,36,37,39,40], and two the pennation angle [7,34]. Regarding muscles, the most evaluated muscle was the rectus femoris [6,7,19,23,24,34,35,36,39,40,42,44,45], which was analyzed in all characteristics mentioned above. Other well-evaluated muscles were the biceps brachii [37,39,41,43,45], vastus intermedius [7,23,35,45], and tibialis anterior [6,37,39,40]; assessments also considered the quadriceps femoris, with joint assessment of rectus femoris and vastus intermedius [6,23,24,36,41]. Finally, the vastus lateralis [7,23,35], diaphragm [34,38], brachioradialis [37], flexor carpi radialis [39], and internal intercostals [34] were mentioned less frequently.

### 3.5. Assessments Used for Functioning Outcomes

Considering the components of the international classification of functioning disability and health (ICF), in the body functions and structures category, the main component assessed was muscle strength. This was mainly evaluated by the Medical Research Council sum-score (MRC-SS), used in 13 articles, also mentioning the assessment of handgrip strength using a dynamometer and other muscle groups using manual dynamometry (handheld). Regarding the activities and participation component, mobility was the main one assessed. The most used instrument (four articles) for this assessment was the ICU Mobility Scale (IMS), followed by the ICU Physical Function Test (PFIT-s) in two articles. Also, ICU Functional Status Scale (FSS-ICU), Short Physical Performance Battery (SPPB), 4 m gait speed test, 6 min walk test, 5-time sit–stand test, balance assessment, Modified Rankin scale, and a Walking ability test named ICF-walking were cited only once each among the articles included. Regarding self-care, two articles used the Barthel Index.

### 3.6. US Assessment Period

Studies presented different protocols in regard to the assessment period. Most underwent the first assessment upon admission, which was considered within 48 h of admission. Only one study used the awakening of participants as a criterion for the first assessment, which occurred on a median of 7 to 9 days after admission [39], and another performed US assessments only after 14 days of hospitalization or at hospital discharge, whichever occurred first [35]. Regarding other muscle assessments with US, assessments were carried out at 2, 3, 5, 7, 10, 14, and 20 days, ICU discharge, and hospital discharge.

The results of the included studies demonstrated a relationship between MT and strength. Reduced MT was associated with muscle weakness in several studies, including reduced MT of the rectus femoris [24,45], vastus intermedius [7,45], vastus intermedius lateral view of the thigh and vastus intermedius + vastus lateralis [23], biceps brachii [41], and quadriceps femoris [23,41]. Regarding CSA, an association has been demonstrated between muscle weakness and reduced CSA in the rectus femoris [42,43,45], vastus intermedius [45], and biceps brachii [43]. Worsening muscle quality (EI) of the vastus intermedius [7], vastus lateralis [35], and rectus femoris [6,23] was also associated with muscle weakness.

Regarding diagnostic accuracy, Zhang et al. [45] found that muscular changes in the MT and CSA of the right rectus femoris and vastus intermedius on both sides showed good diagnostic accuracy for the diagnosis of ICUAW. Formenti et al. [34] reported that the rate of reduction in the pennation angle of the rectus femoris was the best predictor of ICUAW. Finally, Hadda et al. [38] found that a decline in the MT of diaphragm can predict the development of ICUAW.

### 3.7. Relationship between Muscle Changes Assessed by US and Functional Capacity

Regarding mobility, correlations have been reported between mobility difficulties at ICU discharge and reduced MT of the vastus intermedius [7,23], vastus intermedius lateral view of the thigh, rectus femoris + vastus intermedius, vastus lateralis, and vastus lateralis + vastus intermedius [23]. A correlation has also been reported between decreased CSA of the biceps brachii and rectus femoris [43]. Regarding self-care and the ability to perform daily activities, there was a correlation between increased mean echogenicity assessed by Heckmatt score on day 10 and a decreased Barthel index and an increased modified Rankin scale score on day 100 [37].

### 3.8. Meta-Analysis

Nine studies were included in the meta-analysis. In regard to the seven excluded articles, five were excluded because the statistical analysis did not include correlation analyses or they did not allow the conversion of the effect size as mentioned before, one was excluded because it contained only general analysis and it did not specify each muscle result, and one article was excluded because it included only self-care outcomes. Correlation analyses were carried out between the muscular characteristics assessed by US (MT, CSA, and EI) and the functionality outcomes (Muscle Strength and Mobility). In subgroup analyses, correlation analyses were carried out between the muscle and the outcome assessment, considering each muscle characteristic assessed by US (i.e., MT of the rectus femoris and MRC-SS), when possible.

Forest plots and funnel plots of the analyses are shown in Supplementary Materials S2. The results of the moderator analyses (meta-regression) of all analyses carried out are shown in Table 4.

#### 3.8.1. Correlation between MT and Muscle Strength

There is evidence of a correlation between MT and muscle strength (r = 0.20 [0.11; 0.27]; *p* < 0.0001), as shown in Figure S2 (Supplementary Materials S2). The correlation showed low heterogeneity (I^2^ = 27.95%; Q = 49.3488; *p* = 0.0683), but the funnel plot (Figure S23 in Supplementary Materials S2) did not show this, and Egger’s test showed a risk of bias in the studies analyzed (z = 3.2271; *p* = 0.0013). A statistically significant difference was evidenced in the meta-regression analysis with the moderators “Average age of participants” (*p* = 0.0001) and “Time between the first and last US assessment” (*p* < 0.0001), demonstrating the possible influence of these variables on the results found.

Regarding subgroup analyses, investigations were carried out on the correlations between the MT of rectus femoris, vastus intermedius, and vastus lateralis with muscle strength assessed by MRC-SS (Figures S3, S4 and S5 in Supplementary Materials S2, respectively), and between MT of rectus femoris and vastus lateralis with muscle strength assessed by handheld dynamometer (Figures S6 and S7 in Supplementary Materials S2, respectively).

There was no correlation between MT of rectus femoris and muscle strength assessed by MRC-SS (r = 0.06 [−0.24; 0.35]; *p* = 0.69), with moderate heterogeneity (I^2^ = 53.83%; Q = 6.63; *p* = 0.08); however, the funnel plot (Figure S24 in Supplementary Materials S2) and Egger’s test showed a risk of bias in the studies analyzed (z = 2.38; *p* = 0.0171). A correlation was evidenced between MT of vastus intermedius and muscle strength assessed by MRC-SS (r = 0.42 [0.18; 0.61]; *p* = 0.0009), without heterogeneity (I^2^ = 0.00%; Q = 0.98, *p* = 0.6129), with no evidence of risk of bias, according to the funnel plot (Figure S25 in Supplementary Materials S2) and the Egger’s test (z = −0.188, *p* = 0.85). Finally, a correlation was evidenced between MT of vastus lateralis and muscle strength assessed by MRC-SS (r = 0.33 [0.07; 0.55]; *p* = 0.013), with low heterogeneity (I^2^ = 4.48%; Q = 1.9973, *p* = 0.37), with no evidence of risk of bias, according to the funnel plot (Figure S26 in Supplementary Materials S2) and the Egger’s test (z = 0.81 *p* = 0.42).

There was correlation between MT of rectus femoris and muscle strength assessed by handheld dynamometer (r = 0.31 [0.03; 0.55]; *p* = 0.0281), with no heterogeneity (I^2^ = 0.00%; Q = 0.7602; *p* = 0.6838), and the funnel plot (Figure S27 in Supplementary Materials S2) and Egger’s test did not show a risk of bias in the studies analyzed (z = 0.3052; *p* = 0.7602). Finally, there was no correlation between MT of vastus lateralis and muscle strength assessed by handheld dynamometer (r = −0.01 [−0.29; 0.27]; *p* = 0.9440), with no heterogeneity (I^2^ = 0.00%; Q = 2.1096, *p* = 0.3483), with no evidence of risk of bias, according to the funnel plot (Figure S28 in Supplementary Materials S2) and the Egger’s test (z = −1.2265; *p* = 0.2200).

#### 3.8.2. Correlation between CSA and Muscle Strength

There is evidence that there is no correlation between CSA and muscular strength (r = 0.15 [−0.04; 0.33]; *p* < 0.0001), as shown in Figure S8 (Supplementary Materials S2). The correlation showed moderate heterogeneity (I^2^ = 74.64%; Q = 59.3516; *p* < 0.0001), with risk of bias according to the funnel plot (Figure S29 in Supplementary Materials S2) and the Egger’s test (2.2687, *p* = 0.0233). A statistically significant difference was evidenced in the meta-regression analysis with the moderators “Average age of participants” (*p* = 0.0282), as shown in Table 4.

In the subgroup analysis, it was also observed that there is no correlation between CSA of rectus femoris and muscle strength assessed by MRC-SS (r = 0.09 [−0.14; 0.30]; *p* = 0.4517), as shown in Figure S9 (Supplementary Materials S2). The correlation showed moderate heterogeneity (I^2^ = 69.75%; Q = 24.6946; *p* = 0.0018), with a risk of bias according to the funnel plot (Figure S30 in Supplementary Materials S2); however, the Egger’s test did not show a risk of bias in the studies analyzed (z = 1.8489, *p* = 0.0645). It was also observed that there is no correlation between CSA of rectus femoris and muscle strength assessed by handgrip strength dynamometer (r = 0.07 [−0.58; 0.60]; *p* = 0.4517), as shown in Figure S10 (Supplementary Materials S2). The correlation showed high heterogeneity (I^2^ = 91.58%; Q = 23.5312; *p* < 0.0001), with a risk of bias according to the funnel plot (Figure S31 in Supplementary Materials S2) and the Egger’s test (z = 4.8398, *p* < 0.0001).

#### 3.8.3. Correlation between EI and Muscle Strength

There is no evidence of a correlation between EI and muscular strength (r = −0.19 [−0.50; 0.16]; *p* = 0.2760), as shown in Figure S11 (Supplementary Materials S2). The analysis showed high heterogeneity (I^2^ = 77.10%; Q = 26.2294, *p* = 0.0005), with evidence of risk of bias, according to the funnel plot (Figure S32 in Supplementary Materials S2) and Egger’s test (z = 1.9829, *p* = 0.0474). A statistically significant difference was evidenced in the meta-regression analysis with the moderators “Average age of participants” (*p* = 0.0312), as shown in Table 4. In the subgroup analysis, a correlation was observed between EI of rectus femoris and muscle strength assessed by MRC-SS (r = −0.40 [−0.61; −0.13]; *p* = 0.0042), as shown Figure S12 (Supplementary Materials S2). The correlation showed low heterogeneity (I^2^ = 47.85%; Q = 5.77; *p* = 0.12), with no risk of bias according to the funnel plot (Figure S33 in Supplementary Materials S2) and Egger’s test (z = −0.42; *p* = 0.67).

#### 3.8.4. Correlation between MT and Mobility

There is evidence of a correlation between MT and mobility (r = 0.35 [0.19; 0.49]; *p*< 0.0001), as shown in Figure S13 (Supplementary Materials S2). The correlation showed moderate heterogeneity (I^2^ = 69.85%; Q = 68.8467; *p* < 0.0001), with evidence of risk of bias according to the funnel plot (Figure S34 in Supplementary Materials S2); however, the Egger’s test did not show the same evidence (z = 1.7934, *p* = 0.0729). A statistically significant difference was evidenced in the meta-regression analysis with the moderator “Methodological quality of studies” (*p* = 0.0007), demonstrating the possible influence of the quality of studies on the results found. The other moderators analyzed did not show statistical significance, as shown in Table 4.

Regarding subgroup analyses, analyses were carried out on the correlations between the MT of the rectus femoris, vastus intermedius, and vastus lateralis with mobility assessed by the IMS (Figures S14, S15, and S16 in Supplementary Materials S2, respectively). There was no correlation between MT of rectus femoris and mobility assessed by IMS (r = 0.28 [−0.22; 0.66]; *p* = 0.27), with moderate heterogeneity (I^2^ = 72.90%; Q = 7.60; *p* = 0.02), with risk of bias according to the funnel plot (Figure S35 in Supplementary Materials S2); however, the Egger’s test did not show a risk of bias in the studies analyzed (z = 0.16; *p* = 0.87). There was also no correlation between MT of vastus intermedius and mobility assessed by IMS (r = 0.55 [−0.08; 0.87]; *p* = 0.08), with high heterogeneity (I^2^ = 85.85 %; Q = 14.83; *p* = 0.0006), with a risk of bias according to the funnel plot (Figure S36 in Supplementary Materials S2), but the Egger’s test did not show a risk of bias in the studies analyzed (z = 0.27; *p* = 0.78). Finally, a correlation was evidenced between MT of vastus lateralis and mobility assessed by IMS (r = 0.43 [0.13; 0.66]; *p* = 0.0065), with low heterogeneity (I^2^ = 35.68%; Q = 3.07; *p* = 0.21), without risk of bias according to the funnel plot (Figure S37 in Supplementary Materials S2) and the Egger’s test (z = 0.99; *p* = 0.32).

#### 3.8.5. Correlation between CSA and Mobility

There is evidence that there is a correlation between CSA and mobility (r = 0.30 [0.10; 0.47]; *p* = 0.0038), as shown in Figure S17 (Supplementary Materials S2). The correlation showed moderate heterogeneity (I^2^ = 72.36%; Q = 44.0181; *p* < 0.0001), with risk of bias, according to the funnel plot (Figure S38 in Supplementary Materials S2) and the Egger’s test (z = 2.3796, *p* = 0.0173). No statistically significant differences were evident in the meta-regression analyses with moderators, as shown in Table 4. In the subgroup analysis, it was also observed that there is a correlation between the CSA of the rectus femoris and the mobility assessed by IMS (r = 0.38 [0.13; 0.59]; *p* = 0.004), as shown in Figure S18 (Supplementary Materials S2). The correlation showed low heterogeneity (I^2^ = 34.77%; Q = 4.65, *p* = 0.20), with no risk of bias, according to the funnel plot (Figure S39 Supplementary Materials S2) and the Egger’s test (z = −0.06; *p* = 0.95).

#### 3.8.6. Correlation between EI and Mobility

There is evidence of a correlation between EI and mobility (r = −0.29 [−0.53; −0.01]; *p* = 0.0427), as shown in Figure S19 (Supplementary Materials S2). The correlation showed high heterogeneity (I^2^ = 79.42%; Q = 39.89; *p* < 0.0001), with risk of bias, according to the funnel plot (Figure S40 in Supplementary Materials S2) and the Egger’s test (z = −2.3168, *p* = 0.0205). There was not any statistically significant differences evidenced in the meta-regression analyses with moderators, as shown in Table 4. In the subgroup analysis, a correlation was also observed between EI of the rectus femoris and mobility assessed by IMS (r = −0.25 [−0.48; −0.00]; *p* = 0.0499), as shown in Figure S20 (Supplementary Materials S2). The correlation showed no heterogeneity (I^2^ = 0.0%; Q = 0.17; *p* = 0.92), no risk of bias according to the funnel plot (Figure S41 in Supplementary Materials S2) and Egger’s test (z = −0.2; *p* = 0.84).

#### 3.8.7. Sensitivity Analysis

Sensitivity analyses were performed for meta-analyses that showed high heterogeneity. One analysis of a study was excluded when it showed the greatest disparity among results of the included studies. In the correlation between EI and strength, the analysis (VL EI × HHD) [35] was excluded. As a result, the correlation was evident (r = −0.33 [−0.54; −0.09]; *p* = 0.0090), as shown in Figure S21 (Supplementary Materials S2). The correlation showed moderate heterogeneity (I^2^ = 53.14%; Q = 12.6959; *p* = 0.0481), with a risk of bias according to the funnel plot (Figure S42 in Supplementary Materials S2); however, the Egger’s test did not show a risk of bias in the studies analyzed (1.1665, *p* = 0.2434). Regarding the correlation between EI and mobility, an analysis of a study was excluded (VL EI × HHD) [35]. As a result, a correlation was found (r = −0.37 [−0.58; −0.10]; *p* = 0.0070), as shown in Figure S22 (Supplementary Materials S2). The correlation showed moderate heterogeneity (I^2^ = 72.89%; Q = 26.7217; *p* = 0.0004), with risk of bias according to the funnel plot (Figure S43 in Supplementary Materials S2), but not on the Egger’s test (z = −1.5576, *p* = 0.1193).

## 4. Discussion

The present study sought to investigate, through a literature review and meta-analysis, the relationship between muscular characteristics assessed by US, strength, and functional capacity of individuals after ICU discharge. In this study, sixteen articles were included and nine were analyzed in the meta-analysis. This study showed that there is a relationship between muscle changes assessed by US and changes in functioning, including ICUAW and changes in mobility, after admission to the ICU.

Muscle assessment through US is useful for analyzing structural and qualitative changes and the trajectories of these changes in skeletal muscles during the hospitalization period [46]. Despite all the recommendations for the use of US in this assessment, disparities are still observed in research [28,29]. In this systematic review, these disparities were observed, including a variety of equipment with different technological advances used, variety of quantity and time between assessments, image acquisition, and analysis techniques, among other methodological differences. Adding to this, US assessment is evaluator-dependent, which generates great diversity in the results found.

Initially, it is highlighted that the main muscle group evaluated in this study was the quadriceps femoris. The quadriceps femoris is composed of four muscles (rectus femoris, vastus lateralis, vastus intermedius, and vastus medialis) that perform the functions of hip flexion and knee extension and medial and lateral rotation of the hip [47]. The choice of this muscle group for these studies has been justified for a few reasons. They are accessible muscles, with large volume and well-defined anatomical conditions [7]; they can reflect the skeletal muscle mass of the entire body when evaluated by computed tomography [48]; they present inter- and intra-examiner reliability in US assessment [15,16,49]; they have been related to ICUAW, changes in functional capacity, and worse clinical outcomes [6,7]. It is noteworthy that other muscles/muscle groups were evaluated in the original studies included, such as the biceps brachii and tibialis anterior; however, they could not be included in the subgroup analyses due to the insufficient number of studies that analyzed them.

Regarding the muscular characteristics observed in the US assessment, data highlight MT and CSA as the main parameters used in research; they are essential markers for analyzing the quantitative trajectory of peripheral muscle mass [11]. Furthermore, another fundamental parameter is EI, which evaluates muscle quality and is correlated with the quality of muscle contraction [11].

In this systematic review, a relationship between MT and muscle strength and mobility was shown. Regarding muscle strength, in general, conditions were reported that may have influenced this result, such as the age of the participants and the time between muscle assessments using US. It is understood that these findings reflect the sociodemographic profile of participants included in original studies, including adults over 18 years of age, but the impacts of muscular changes are more prevalent and intense in the older population. Furthermore, the time between US assessments in the original studies varied between three and twenty days, which may have influenced the amount of altered MT, even considering that there is a more significant reduction in muscle mass in the first seven days of admission to the ICU [50].

However, when rectus femoris, vastus intermedius, and vastus lateralis muscles were associated with muscular strength, as well as the latter with mobility, the results presented were more robust. It is noteworthy that the study by Hayes et al. [23] presented significant results of the correlation between the MT of vastus intermedius and vastus lateralis, both individually and together, with strength and mobility outcomes. These results occurred mainly when the US evaluation of these muscles occurred within 20 days of ICU stay, in patients who needed extracorporeal membrane oxygenation (ECMO) [23].

Regarding CSA, a relationship with mobility was evidenced, especially with the rectus femoris. It is noteworthy that the study carried out by Borges and Soriano [42] showed a negative correlation between CSA and muscle strength. This fact is due to how muscle strength was analyzed, considering that this was observed through the difference between measurements when patients woke up and when they were discharged, in which a gradual increase in strength was reported. However, the authors report that even with increased strength, around 25% demonstrate signs of ICUAW when assessed using the MRC-SS and 1/3 of patients using handgrip dynamometry. Furthermore, they report as a limitation that the tests depend on the patients’ will and level of consciousness, which may have impacted the results; however, strategies have been reported to reduce this influence [42]. Also, in regards to mobility, the study carried out by Andrade-Junior et al. [36] had the same correlation direction, which occurred due to the US assessment being performed only once, at the time of discharge.

Finally, concerning to EI, associations demonstrated that the higher the mean value of the gray scale in the muscle assessment, in other words, the hyperechoic image, the worse the quality of the muscle and, consequently, the worse the muscle strength and mobility, especially for the rectus femoris. Because of the great disparities, meta-analyses showed high heterogeneity; however, when subjected to sensitivity analysis, the results were valid. Considering the few studies that included the assessment of EI, EI measurements of the rectus femoris and vastus intermedius were those that brought significant correlations with the outcomes in the original studies [7,23].

Overall, the limited amount of research included in this study had moderate to good quality. The general meta-analyses showed significant correlations with low to high heterogeneity for the muscular strength outcome and moderate to high heterogeneity for the mobility outcome. In general, analyses of the muscular strength outcome are more widespread in the literature and were present in around 90% of the included studies. The prevalence of ICUAW is high in the population discharged from the ICU, with evidence of a prevalence of 48% (CI 39–56%) in a recent systematic review with meta-analysis [50]. However, no other assessments of limitations that could be associated with the muscular changes assessed were evidenced.

This also reflects the interest in evaluating the outcome related to body function, based on a biomedical model, disregarding other factors that may reflect on individuals’ ability to engage in activities and engage in social participation [51]. In this aspect, Puthucheary and Hart [52] already reported that the decrease in muscle mass could be associated with a reduction in muscle strength and functional capacity; however, they stated that more studies would still be needed with the expectation of predicting the functional outcome of patients, and thus, being able to direct intra- and post-hospital interventions [52].

Although this review included studies that assessed the ability to perform functional mobility, only the studies carried out by Tanaka et al. [44] and Klawitter et al. [37] evaluated the correlation with the ability to perform self-care. Tanaka et al. [44] analyzed this correlation with the MT of the rectus femoris, in which there was no association; however, on the 14th day, the median of the Barthel Index was 0.0 (0.0–37.5), demonstrating the high rate of disability of study participants [44]. However, Klawitter et al. [37] found that there was a correlation between an increased EI assessed by the Heckmatt score on day 10 and muscular weakness assessed by MRC, and higher dependence on activities of daily living assessed by the Barthel Index and modified Rankin scale on day 100 after study inclusion.

It is important to highlight that, even though these muscles were evaluated in the original studies, analyses were not carried out considering the changes over the whole body, but their individual results in the majority of the studies. It should be noted that there are not enough data for the estimation of measurements of a single muscle to be considered adequate to demonstrate muscular changes throughout the whole body [10].

In this sense, two observations must be made. The first is that muscular changes can occur in different periods and conditions for each muscle/muscle group; the second is that assessment of a muscle/muscle group is not sufficient to reflect muscular changes throughout the whole body. Taking this into consideration, only the study carried out by Klawitter et al. [37] performed analyses using the global mean of echointensity of rectus femoris, tibialis anterior, biceps brachii, and brachioradialis. They were assessed on days 3 and 10 after ICU admission, using two assessment methods (gray scale on ImageJ and the Heckmatt Scale).

Therefore, studies need to be conducted to verify this relationship between muscular changes in a muscle or individual muscle assessments with muscular changes in the entire body. They must also evaluate muscular changes, quantitatively and qualitatively, during ICU stay, correlating their effects with the functioning of individuals after ICU and hospital discharge. Furthermore, the methodological precautions already described by other authors must be considered, including standardization in the training of evaluators, in the location of points of interest for the assessment, in the use of equipment, and in the acquisition and analysis of images [11,28].

## 5. Conclusions

Taking into consideration all the aspects presented, the study presented evidence that there is a correlation between the muscular characteristics assessed by US and functioning outcomes. The specificities of the muscular characteristics analyzed (MT, CSA, and EI) must be considered, as well as the muscles evaluated, considering the correlations between these (rectus femoris, vastus intermedius, and vastus lateralis) and the outcomes of muscular strength and mobility. It is noteworthy that other muscular characteristics and muscles did not present sufficient evidence for quantitative analysis.

## Figures and Tables

**Table 1:** Summary of information retrieved from the 16 studies included in this systematic review.

Author, Year of Publication	N	Main Objective	Muscle Characteristics Assessed by US	Assessments of Functioning Outcomes (ICF Domain)	Results
Parry, El-Ansary, et al., 2015 [7]	22	To determine the rate of muscle loss during the first 10 days of ICU admission and the relationship between US muscle assessment, strength, and functioning at ICU awakening and ICU discharge.	The MT and EI of the vastus intermedius and rectus femoris, MT of the vastus lateralis, subcutaneous tissue, CSA of the rectus femoris and vastus lateralis pennation angle were assessed after admission to the ICU, days 3, 5, 7, and 10, upon awakening and at discharge from the ICU.	Strength was assessed using the MRC and function assessed using the PFIT-s and IMS upon awakening and at discharge from the ICU. (Body structure and function; Activity).	There was a strong correlation between MT of the vastus intermedius and EI, and measures of strength and function. There was a strong correlation between MT of the vastus intermedius and functional outcomes measured by PFIT-s and IMS at ICU discharge.
Witteveen, Sommers, et al., 2017 [39]	71	To investigate the diagnostic accuracy of quantitative neuromuscular US for the diagnosis of ICUAW.	MT and EI of the biceps brachii, anterior tibialis, and median nerve muscles, and the rectus femoris muscle, flexor carpi radialis muscle were evaluated at the time of the patient’s awakening.	Clinical assessment of muscle strength was performed using the MRC (Body Function and Structure)	The accuracy of US for diagnosing ICUAW is poor when assessed upon awakening (median 7 to 9 days after ICU admission).
Connolly, Maddocks, et al., 2018 [40]	16	To analyze changes in muscle strength in patients admitted to the ICU and their relationship with the assessment of peripheral skeletal muscle architecture.	MT, CSA, EI, and subcutaneous tissue thickness of the rectus femoris and tibialis anterior muscles were asessed.	Ankle dorsiflexor muscle strength was assessed using Ankle Dorsiflexor Muscle Force induced by supramaximal electrical stimulation of the common peroneal nerve using an extensometer attached to the lower part of the device platform. (Body Function and Structure)	Positive correlations were observed between anterior tibialis CSA and rectus femoris CSA in test 1. Only 100 HzAD and tibialis anterior CSA demonstrated a significant correlation in test 2.
Hadda, Kumar, et al., 2018 [41]	70	To describe trends in MT loss in the arm and thigh (assessed by US) and determine the relationship between MT loss and in-hospital and post-discharge outcomes.	MT measurements of the biceps brachii (including the coracobrachialis) and quadriceps femoris were performed on days 1, 3, 5, 7, 10, and 14 and subsequently weekly, until the patient’s discharge or death.	Clinical assessment of muscle strength was performed using the MRC. (Body Function and Structure).	There was a correlation between having ICUAW and a greater decline in MT biceps brachii and quadriceps femoris on days 3, 5, and 7.
Hayes, Holland, et al., 2018 [23]	25	Describe muscle changes from day 1 to day 10 and the relationship between ultrasound measurements and measurements of muscle strength and level of mobility on days 10 and 20.	Measurements of the anterior thigh: EI, CSA, and MT of the rectus femoris, MT of the vastus intermedius and total MT of quadriceps femoris (rectus femoris + vastus intermedius). Lateral thigh measurements: MT of the vastus lateralis, MT of the vastus intermedius, and total MT (vastus lateralis + vastus intermedius).	Muscle strength was assessed using MRC and isometric peak knee extension strength using the Nicholas Manual Muscle Tester. Mobility in the ICU was assessed using the IMS (Body function and structure; Activity).	On day 10, there was only a correlation between rectus femoris IE and MRC. On day 20, there was a correlation between MT of the vastus intermedius anterior and lateral thigh, rectus femoris + vastus intermedius, vastus lateralis and vastus lateralis + vastus intermedius and mobility changes; MT of rectus femoris + vastus intermedius, lateral vastus intermedius of the thigh and EI of rectus femoris and peak knee extension strength; MT vastus lateralis + vastus intermedius and MRC.
Palakshappa et al., 2018 [24]	19	To describe the relationship between CSA of rectus femoris and MT of quadriceps femoris with muscle strength and function of septic patients.	CSA of rectus femoris and MT of quadriceps femoris.	Strength was assessed using the MRC and function assessed using the PFIT-s on day 7. (Body structure and function; Activity).	There was a statistically moderate correlation between MRC score and changes of RF-CSA during first seven days of critical illness. Correlation between changes of RF-CSA and PFIT-s, and changes of quadriceps MT with MRC and PFIT-s were not statistically significant.
Berry et al., 2019 [35]	13	To analyze the relationship between MT, EI, and muscle strength in patients with acute respiratory failure.	MT and EI of rectusfemoris, vastus lateralis, and vastus medialis.	Knee extensor strength was assessed by a handheld dynamometer.	There was a statistical correlation between knee extension strength and vastus lateralis EI when controlling for age, body mass index, and fluid intake.
Borges, Soriano, 2019 [42]	37	To evaluate the association between the CSA of the rectus femoris obtained by US and muscle strength in septic patients.	CSA of the rectus femoris was assessed. The assessments began on the second day of hospitalization in the ICU, with the rest being performed on the following days: ‘‘4’’, ‘‘6’’, ‘‘ICU discharge’’, and ‘‘hospital discharge’’.	Assessment of muscle strength using MRC and dynamometry to assess handgrip strength. (Body Function and Structure)	There was an association between CSA of rectus femoris and clinical assessments of muscle strength.
Mayer, Thompson Bastin, et al., 2020 [6]	41	To analyze whether muscle changes during ICU admission are associated with or predict the diagnosis of ICUAW and physical function at hospital discharge	MT, CSA, and EI of the rectus femoris and anterior tibialis and MT of the quadriceps femoris (rectus femoris + vastus intermedius) on days 1, 3, 5, and 7.	Strength was assessed by MRC, handheld dynamometry of the right knee extensors, ankle dorsiflexors and handgrip. Muscle power was evaluated using a linear potentiometer. Physical function was assessed using the 5× Sit-to-Stand Test, the SPPB battery, the 6 min walk test, and the CFS (Body Function and Structure; Activity).	There was no significant correlation between changes in MT, CSA, and EI and the outcomes of strength, muscle power, and mobility. However, muscle power at discharge from the ICU, age, and length of stay in the ICU were associated with performance in the 5× Sit and Stand Test at hospital discharge.
Nakanishi, Oto, et al., 2020 [43]	64	To investigate whether upper limb muscular atrophy was associated with in-hospital mortality. Secondly, the association between upper limb muscular atrophy and physical functions at ICU discharge.	The CSA of the biceps brachii and rectus femoris was assessed at admission and on days 3, 5, and 7.	Muscle strength was assessed using MRC and handgrip dynamometry. Functioning was assessed using the IMS and FSS-ICU. (Body Function and Structure; Activity).	A correlation was found between the MRC score, handgrip strength and FSS-ICU with the reduction in CSA of the biceps brachii and rectus femoris.
Andrade-Junior et al., 2021 [36]	32	To analyze the evaluation of acute muscle loss and its influence onfunctioning of critically ill COVID-19 patients.	MT of the quadriceps femoris, CSA and EI of the rectus femoris.	Muscle strength was assessed using MRC and handgrip dynamometry. Functional capacity was assessed using the IMS and a Walking ability test called ICF-walking. (Body Function and Structure; Activity).	There was a fair correlation between rectus femoris CSA and ICF-walking. There was no correlation between the rectus femoris CSA and MT of quadriceps femoris with handgrip strength.
Hadda, Kumar, et al., 2021 [38]	70	To analyze the relationship between diaphragm thickness assessed by ultrasonography and the outcomes of septic patients.	MT of diaphragm during inspiration and expiration	Muscle strength was assessed using MRC (Body Function and Structure).	Decline in MT of diaphragm can predict the development of ICUAW.
Tanaka, Yamada, 2021 [44]	8	To evaluate changes in the MT of the rectus femoris muscle in patients with septic shock in intensive care and verify the correlation between MT and the ability to perform ADLs.	Rectus femoris MT was assessed on admission and repeated every 2 days until the 13th day. The final observation was carried out on the 14th day of hospitalization.	The ability to perform ADLs was assessed using the Barthel Index on the 14th and 30th day of hospitalization. (Activity).	There was no significant correlation between MT of rectus femoris and the ability to perform ADLs on the 14th and 30th day of hospitalization.
Zhang, Wu, et al., 2021 [45]	37	To analyze the diagnostic accuracy of changes in MT and CSA for the diagnosis of ICUAW	MT and CSA of the biceps brachii, vastus intermedius, and rectus femoris muscles were aseessed on admission, days 4, 7, 10, and discharge from the ICU.	Muscle strength was assessed by the MRC on the 10th day after admission to the ICU. (Body Function and Structure).	A threshold of 15% for ΔMT and a threshold of 12% for ΔCSA of the lower limb muscles showed good diagnostic accuracy for the diagnosis of ICUAW, especially on the right side. Changes in muscle ΔMT and ΔCSA of the lower limbs had diagnostic validity close to SOFA and APACHE II scores at the time of ICU admission.
Formenti, Giorgis, et al., 2022 [34]	50	To evaluate changes in respiratory and peripheral muscles during a long period of ICU stay (>7 days) and analyze the earlier predictive value in the diagnosis of ICUAW, compared to MRC.	The MT of the rectus femoris, diaphragm, and internal intercostals was evaluated; also, CSA and pennation angle of the right rectus femoris were assessed. This was performed on admission (up to 48 h) and repeated on days 3 and 7.	Clinical assessment of muscle strength was performed using the MRC. (Body Function and Structure).	There was an association between the reduction in the pennation angle of the rectus femoris muscle and ICUAW.
Klawitter et al., 2022 [37]	38	To compare the qualitative and quantitative assessments of EI to identify ICUAW.	EI of biceps brachii, brachioradialis, rectus femoris, and tibialis anterior using the Heckmatt Scale and Quantitative Greyscale Analysis of Muscle Echogenicity with ImageJ	Clinical assessment of muscle strength was performed using the MRC on day 3 and day 10. To assess functional outcomes, Barthel index and the modified Rankin scale 100 days after study inclusion. (Body Function and Structure; Activity).	There was a correlation between an increased mean Heckmatt score on day 10, and decreased MRC, and a decreased BI and an increased modified Rankin scale on day 100 after study inclusion.

ADLs: activities of daily living; APACHE II: Acute Physiology and Chronic Health Evaluation II; CFS: Clinical Frailty Scale; CSA: cross-sectional area; ECMO: extracorporeal membrane oxygenation; EI: echogenicity; FSS-ICU: Functional Status Scale for ICU; ICU: intensive care unit; ICUAW: intensive care unit-acquired weakness; IMS: ICU mobility scale; MRC: Medical Research Council; MT: muscle thickness; PFIT-s: physical function in intensive care test; SOFA: sequential organ failure assessment; SPPB: Short Physical Performance Battery; US: ultrasonography.

**Table 2:** Qualitative assessment of studies—The Agency for Healthcare Research and Quality scale (AHRQ).

Author, Year of Publication	Study Design	I	II	III	IV	V	VI	VII	VIII	IX	X	XI	Overall
Witteveen, Sommers, et al., 2017 [39]	Cross-sectional observational	0	1	1	1	1	1	1	1	1	1	1	10/11

**Table 3:** Qualitative assessment of studies—Newcastle-Ottawa Quality Assessment Scale (NOS scale).

Author, Year of Publication	Study Design	Selection	Comparability	Exposure	Overall
Parry, El-Ansary, et al., 2015 [7]	COHORT	2	2	2	6/9
Connolly, Maddocks, et al., 2018 [40]	COHORT	2	2	1	5/9
Hadda, Kumar, et al., 2018 [41]	COHORT	3	2	2	7/9
Hayes, Holland, et al., 2018 [23]	COHORT	3	2	3	8/9
Palakshappa et al., 2018 [24]	COHORT	3	2	3	8/9
Berry et al., 2019 [35]	COHORT	2	2	2	6/9
Borges, Soriano, 2019 [42]	COHORT	2	2	2	6/9
Mayer, Thompson Bastin, et al., 2020 [6]	COHORT	3	2	2	7/9
Nakanishi, Oto, et al., 2020 [43]	COHORT	2	2	3	7/9
Andrade-Junior et al., 2021 [36]	COHORT	3	2	2	7/9
Hadda, Kumar, et al., 2021 [38]	COHORT	2	2	3	7/9
Tanaka, Yamada, 2021 [44]	COHORT	2	2	2	6/9
Zhang, Wu, et al., 2021 [45]	COHORT	3	2	3	8/9
Formenti, Giorgis, et al., 2022 [34]	COHORT	3	2	3	8/9
Klawitter et al., 2022 [37]	COHORT	3	2	3	8/9

**Table 4:** Results of moderator meta-regression analyses.

Correlation	Moderator
MT × strength	MAP: *p* = 0.0001 MQS: *p* = 0.7334 DUS: *p* < 0.0001
CSA × strength	MAP: *p* = 0.0282 MQS: *p* = 0.5077 DUS: *p* = 0.4687
EI × strength	MAP: *p* = 0.0312 MQS: *p* = 0.3359 DUS: *p* = 0.1730
MT × Mobility	MAP: *p* = 0.2096 MQS: *p* = 0.0007 DUS: *p* = 0.6988
CSA × Mobility	MAP: *p* = 0.4789 MQS: *p* = 0.1773 DUS: *p* = 0.4572
EI × Mobility	MAP: *p* = 0.8162 MQS: *p* = 0.1188 DUS: *p* = 0.9253

MAP: mean age of participants; MQS: methodological quality of studies; DUS: days between US.

## Data Availability

The authors confirm that the data supporting the findings of this study are available within the article and its Supplementary Materials.

## Supplementary Materials

The following supporting information can be downloaded at: https://www.mdpi.com/article/10.3390/ijerph21070908/s1, Supplementary Material S1: Databases search strategies; Supplementary Material S2: Meta-analysis plots.

## Registration of Reviews

Registered in PROSPERO database with registration CDR42022361643.
