# Supplementary material for: Impact of Muscle Changes Assessed by Ultrasonography on Muscle Strength and Functioning after ICU Discharge: A Systematic Review with Meta-Analysis
Source: Int J Environ Res Public Health. 2024 Jul 11;21(7):908. doi: 10.3390/ijerph21070908 (PMC11276795; doi:10.3390/ijerph21070908)

Supplementary Material S1: Databases search strategies

- **CINAHL**

(Muscle OR "Intensive care unit acquired weakness") AND (Ultrasonography or Ultrasound) AND ("Intensive care unit" OR "critical care"); Filters: all adult

- **EMBASE**

'observational study'/de AND ([adult]/lim OR [aged]/lim OR [middle aged]/lim OR [very elderly]/lim OR [young adult]/lim) ('muscle'/exp OR muscle OR 'intensive care unit acquired weakness'/exp OR 'intensive care unit acquired weakness') AND ('ultrasonography'/exp OR ultrasonography OR 'ultrasound'/exp OR ultrasound) AND ('intensive care unit'/exp OR 'intensive care unit' OR 'critical care'/exp OR 'critical care')

- **LILACS**

(Muscle OR "Intensive care unit acquired weakness") AND (Ultrasonography or Ultrasound) AND ("Intensive care unit" OR "critical care"); Filter: observational study

- **PubMed**

(Muscle OR "Intensive care unit acquired weakness") AND (Ultrasonography or Ultrasound) AND ("Intensive care unit" OR "critical care") Filters: Observational Study, English, Portuguese, Spanish, Adult: 19+ years, Humans

(("muscle s"[All Fields] OR "muscles"[MeSH Terms] OR "muscles"[All Fields] OR "muscle"[All Fields] OR "Intensive care unit acquired weakness"[All Fields]) AND ("diagnostic imaging"[MeSH Subheading] OR ("diagnostic"[All Fields] AND "imaging"[All Fields]) OR "diagnostic imaging"[All Fields] OR "ultrasonography"[All Fields] OR "ultrasonography"[MeSH Terms] OR "ultrasonographies"[All Fields] OR ("diagnostic imaging"[MeSH Subheading] OR ("diagnostic"[All Fields] AND "imaging"[All Fields]) OR "diagnostic imaging"[All Fields] OR "ultrasound"[All Fields] OR "ultrasonography"[MeSH Terms] OR "ultrasonography"[All Fields] OR "ultrasonics"[MeSH Terms] OR "ultrasonics"[All Fields] OR "ultrasounds"[All Fields] OR "ultrasound s"[All Fields])) AND ("Intensive care unit"[All Fields] OR "critical care"[All Fields])) AND ((observationalstudy[Filter]) AND (humans[Filter]) AND (english[Filter] OR portuguese[Filter] OR spanish[Filter]) AND (alladult[Filter])

- **Science Direct**

(Muscle OR "Intensive care unit acquired weakness") AND (Ultrasonography or Ultrasound) AND ("Intensive care unit" OR "critical care") - Subscribed journals, Research articles.

- **SCOPUS**

( TITLE-ABS-KEY ( muscle ) OR TITLE-ABS-KEY ( "Intensive care unit acquired weakness" ) AND TITLE-ABS-KEY ( ultrasonography ) OR TITLE-ABS-KEY ( ultrasound ) AND TITLE-ABS-KEY ( "critical care" ) OR TITLE-ABS-KEY ( "Intensive care unit" ) ) AND PUBYEAR > 1989

AND ( LIMIT-TO ( DOCTYPE , "ar" ) ) AND ( LIMIT-TO ( LANGUAGE , "English" ) OR LIMIT-TO ( LANGUAGE , "Spanish" ) OR LIMIT-TO ( LANGUAGE , "Portuguese" ) )

- **Web of Science**

(Muscle OR "Intensive care unit acquired weakness") AND (Ultrasonography or Ultrasound) AND ("Intensive care unit" OR "critical care"). Filter: observational study

## Supplementary Material S2: Meta-analysis plots

Figure S2: Forest plot of the correlation between MT and muscle strength

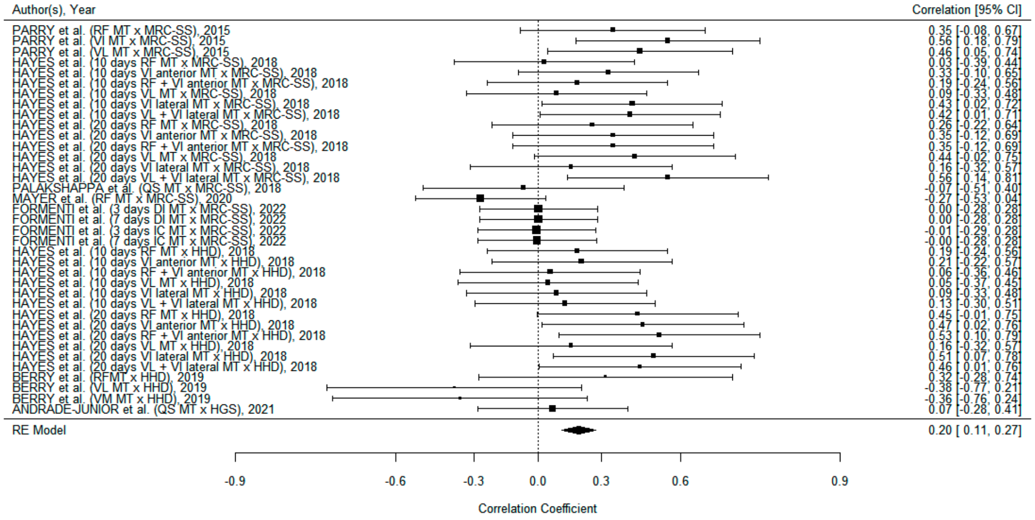


DI: Diaphragm; IC: Parasternal intercostal; MT: Muscle thickness; RF: Rectus Femoris; VI: Vastus Intermedius; VL: Vastus Lateralis; QS: Quadriceps Femoris; MRC-SS: Medical Research Council Sum Score; HHD: Hand Held Dynamometer; HGS: Handgrip strength dynamometer.

Figure S3: Forest plot of the correlation between MT of rectus femoris and muscle strength

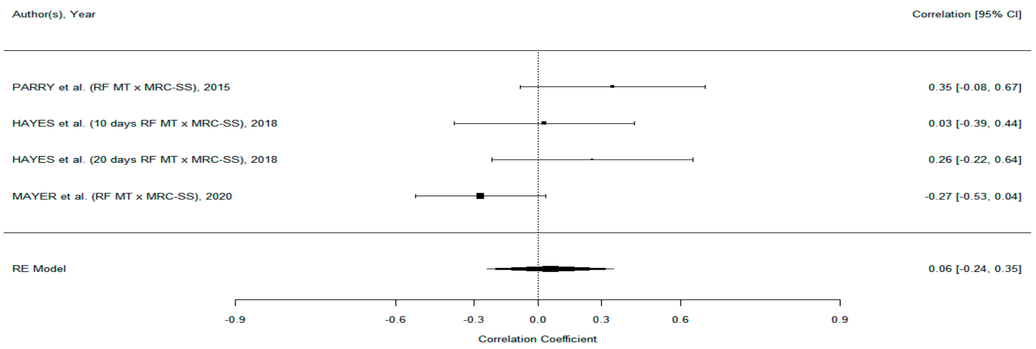


MT: Muscle thickness; RF: Rectus Femoris; MRC-SS: Medical Research Council Sum Score.

Figure S4: Forest plot of the correlation between MT of vastus intermedius and muscle strength assessed by MRC-SS

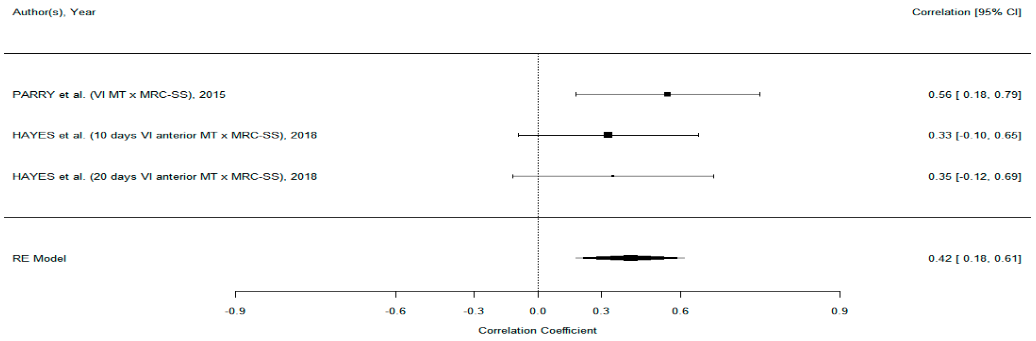


MT: Muscle thickness; VI: Vastus Intermedius; MRC-SS: Medical Research Council Sum Score.

Figure S5: Forest plot of the correlation between MT of vastus lateralis and muscle strength assessed by MRC-SS

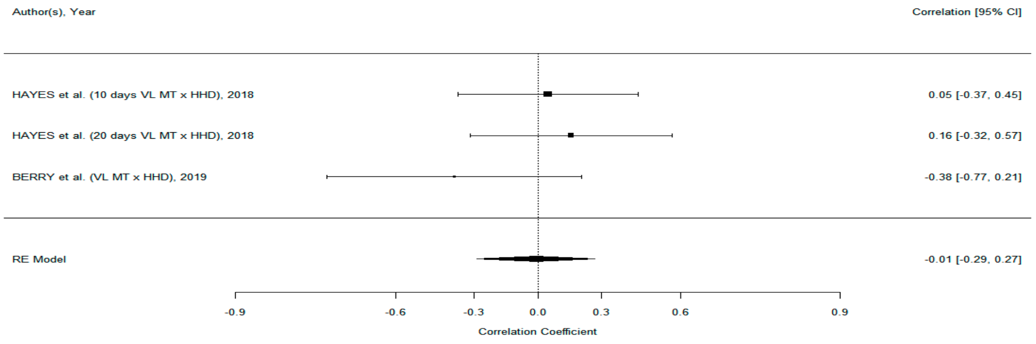


MT: Muscle thickness; VL: Vastus Lateralis; MRC-SS: Medical Research Council Sum Score.

Figure S6: Forest plot of the correlation between MT of vastus intermedius and muscle strength assessed by HHD

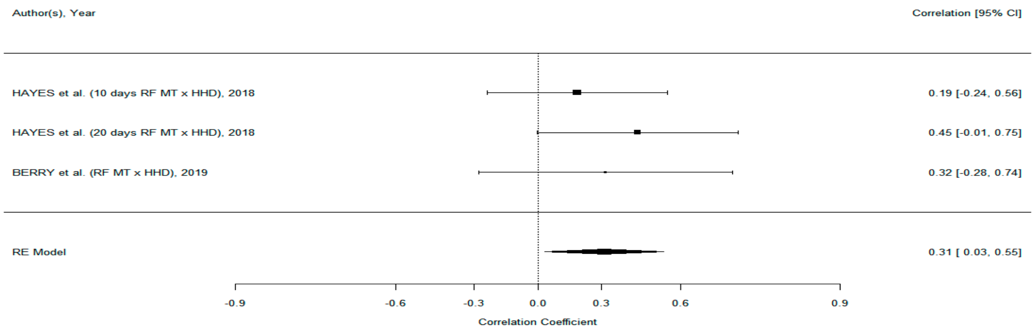


MT: Muscle thickness; RF: Rectus Femoris; HHD: Handheld dynamometer.

Figure S7: Forest plot of the correlation between MT of vastus lateralis and muscle strength assessed by HHD

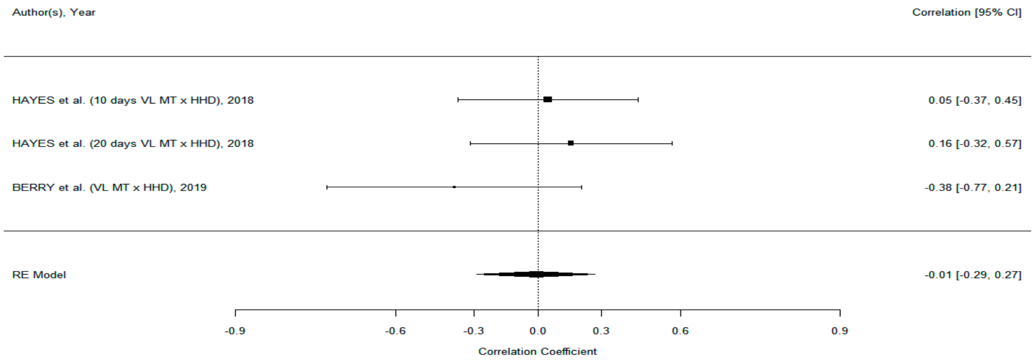


MT: Muscle thickness; VL: Vastus Lateralis; HHD: Handheld dynamometer.

Figure S8: Forest plot of the correlation between CSA and muscle strength

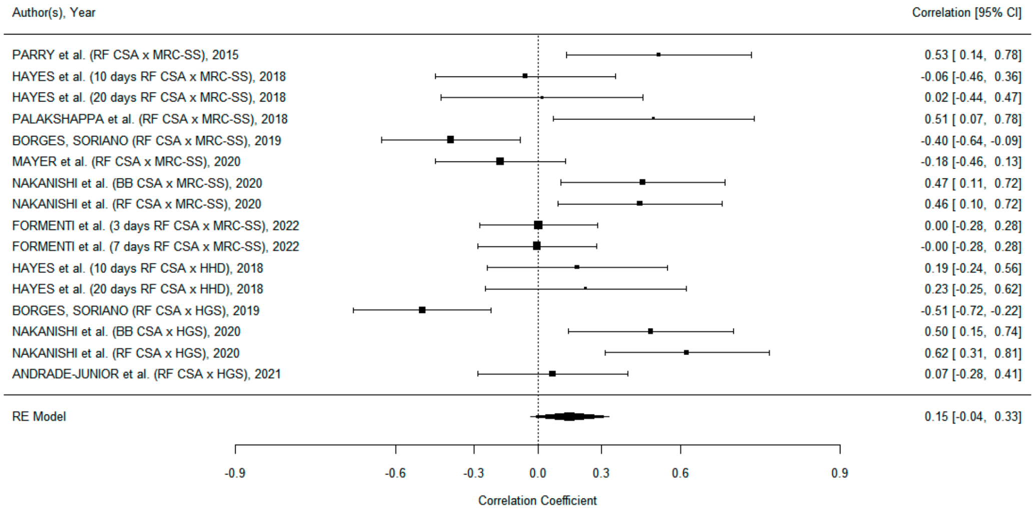


CSA: Cross-sectional area; RF: Rectus Femoris; BB: Biceps Brachii; MRC-SS: Medical Research Council Sum Score; HHD: Hand Held Dynamomenter; HGS: Handgrip strength

Figure S9: Forest plot of the correlation between CSA of rectus femoris and muscle strength assessed by MRC-SS

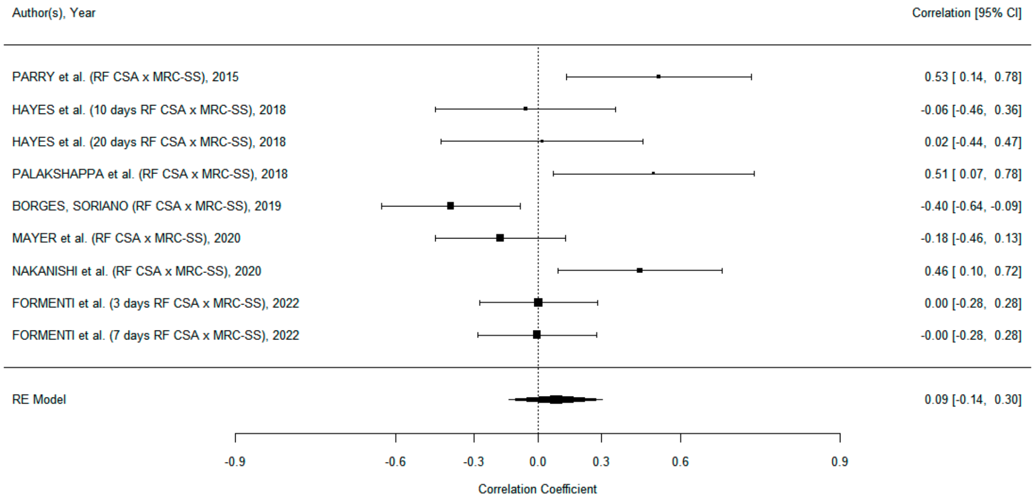


CSA: Cross-sectional area; RF: Rectus Femoris; MRC-SS: Medical Research Council Sum Score.

Figure S10: Forest plot of the correlation between CSA of rectus femoris and muscle strength assessed by HGS

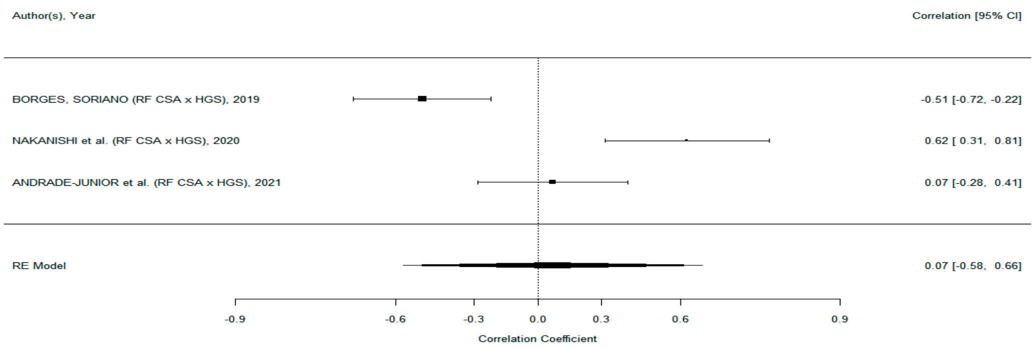


CSA: Cross-sectional area; RF: Rectus Femoris; HGS: Handgrip strength.

Figure S11: Forest plot of the correlation between EI and muscle strength

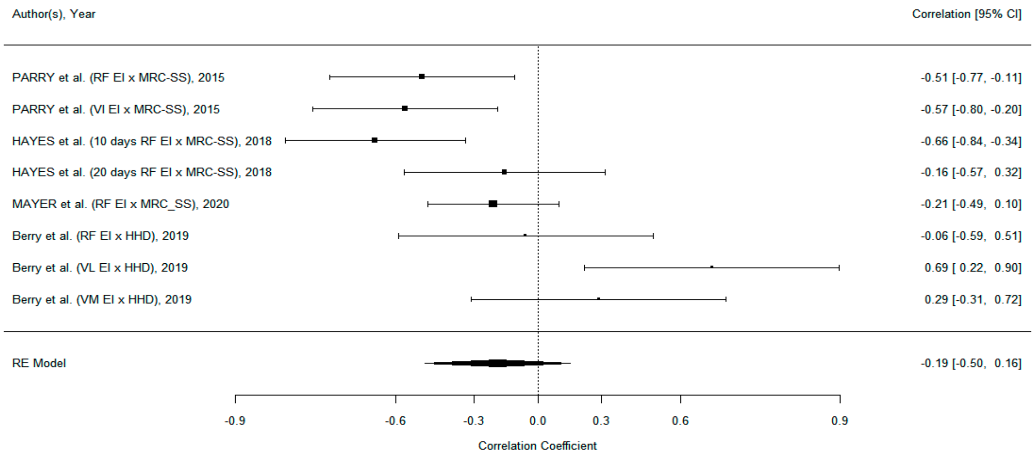


EI: Echointensity; RF: Rectus Femoris; VI: Vastus Intermedius; MRC-SS: Medical Research Council Sum Score; HHD: Handheld dynamometer.

Figure S12: Forest plot of the correlation between EI of rectus femoris and muscle strength assessed by MRC-SS

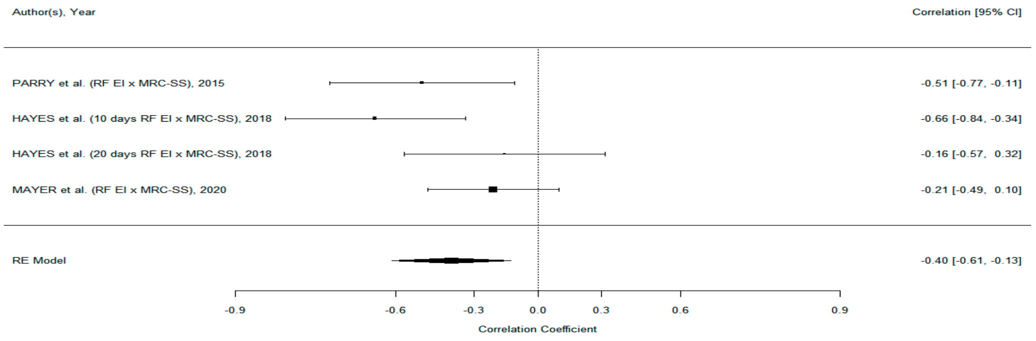


EI: Echo intensity; RF: Rectus Femoris; MRC-SS: Medical Research Council Sum Score.

Figure S13: Forest plot of the correlation between MT and mobility

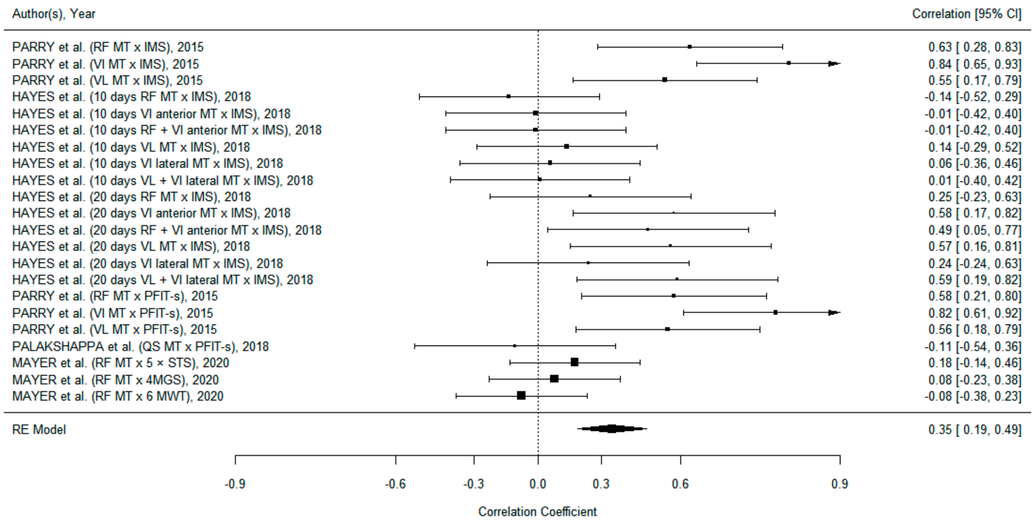


MT: Muscle thickness; RF: Rectus Femoris; VI: Vastus Intermedius; VL: Vastus Lateralis; QS: Quadriceps femoris; IMS: ICU mobility scale; PFIT-s: Physical Function in Intensive Care Test; 5 x STS: Five Times Sit to Stand Test; 4MGS: 4 meter gait speed test; 6MWT: 6-minute walk test.

Figure S14: Forest plot of the correlation between MT of rectus femoris and mobility

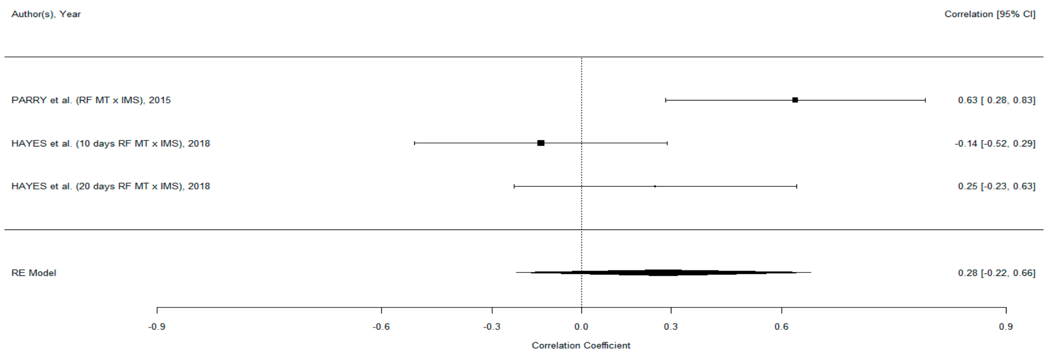


MT: Muscle thickness; RF: Rectus Femoris; IMS: ICU Mobility Scale.

Figure S15: Forest plot of the correlation between MT of vastus intermedius and mobility

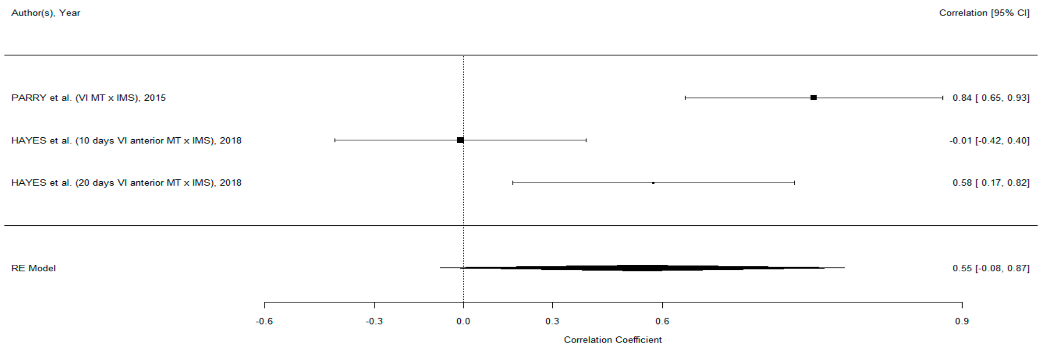


MT: Muscle thickness; VI: Vastus Intermedius; IMS: ICU Mobility Scale.

Figure S16: Forest plot of the correlation between MT of vastus lateralis and mobility

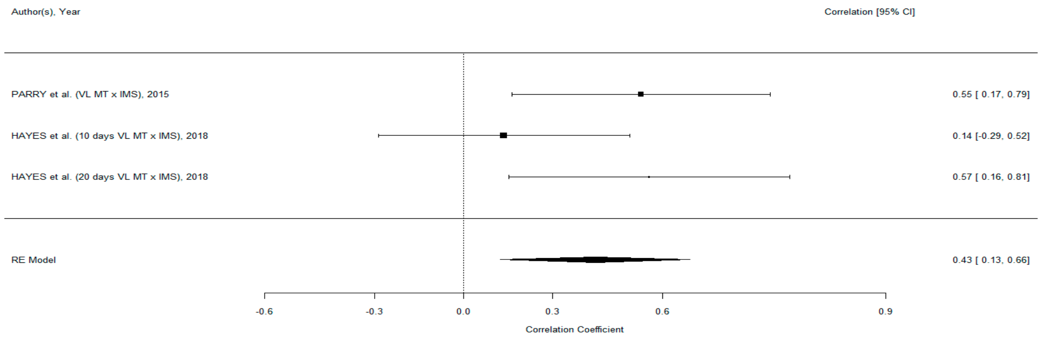


MT: Muscle thickness; VL: Vastus Lateralis; IMS: ICU Mobility Scale.

Figure S17: Forest plot of the correlation between CSA and mobility

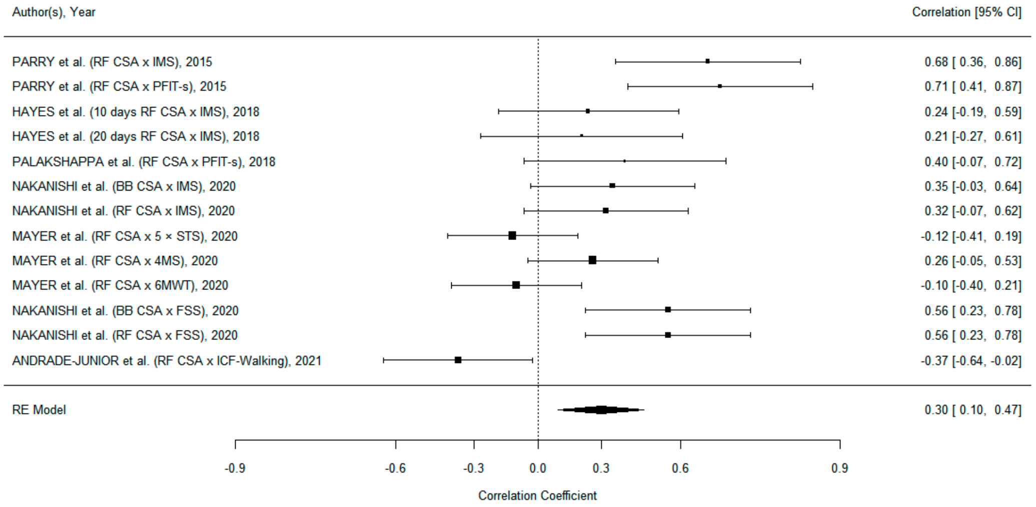


CSA: Cross-sectional area; RF: Rectus Femoris; BB: Biceps Brachii; IMS: ICU mobility scale; PFIT-s: Physical Function in Intensive Care Test; 5 x STS: Five Times Sit to Stand Test; 4MGS: 4 meter gait speed test; 6MWT: 6-minute walk test; FSS: Functional Status Scale for ICU.

Figure S18: Forest plot of the correlation between CSA of rectus femoris and mobility

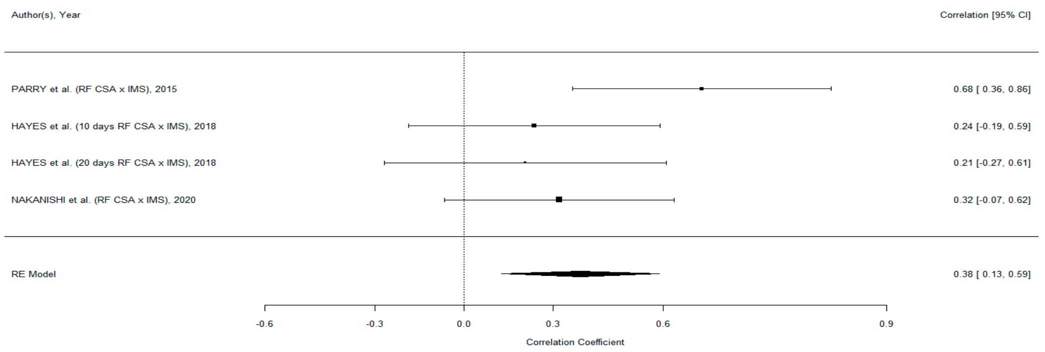


CSA: Cross-sectional area; RF: Rectus Femoris; IMS: ICU mobility scale.

Figure S19: Forest plot of the correlation between EI and mobility

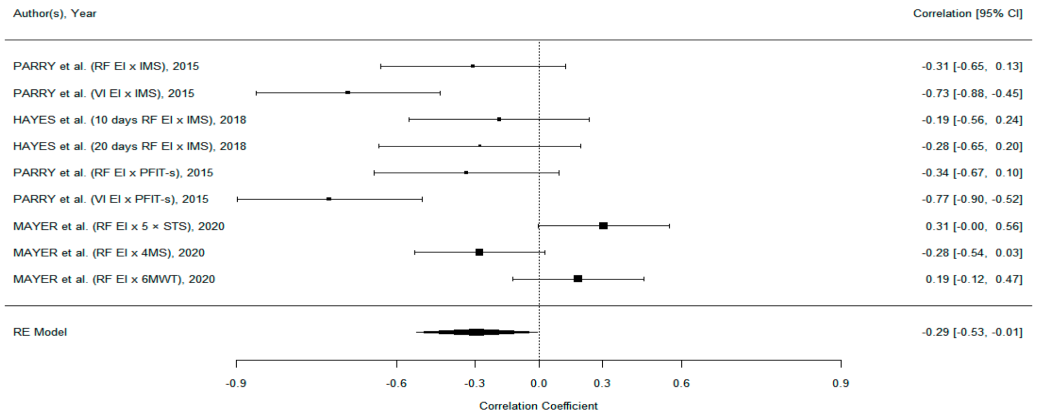


EI: Echo intensity; RF: Rectus Femoris; VI: vastus intermedius; IMS: ICU mobility scale; PFIT-s: Physical Function in Intensive Care Test; 5 x STS: Five Times Sit to Stand Test; 4MGS: 4 meter gait speed test; 6MWT: 6-minute walk test.

Figure S20: Forest plot of the correlation between EI of rectus femoris and mobility

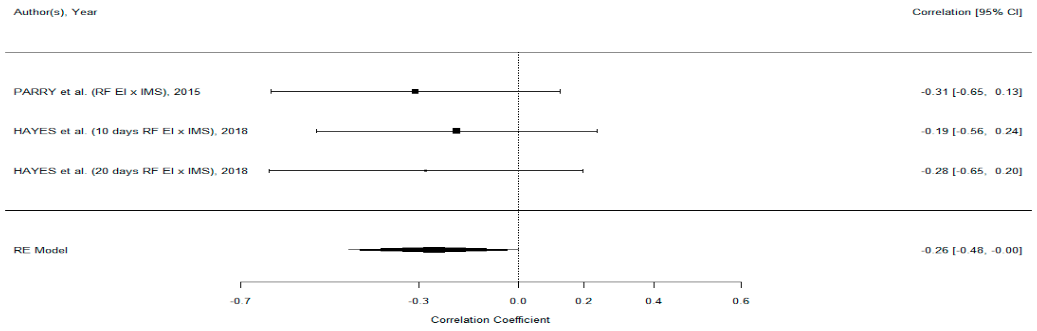


EI: Echo intensity; RF: Rectus Femoris; IMS: ICU mobility scale;

Figure S21: Forest plot of the sensitivity analysis of the correlation between EI and muscle strength

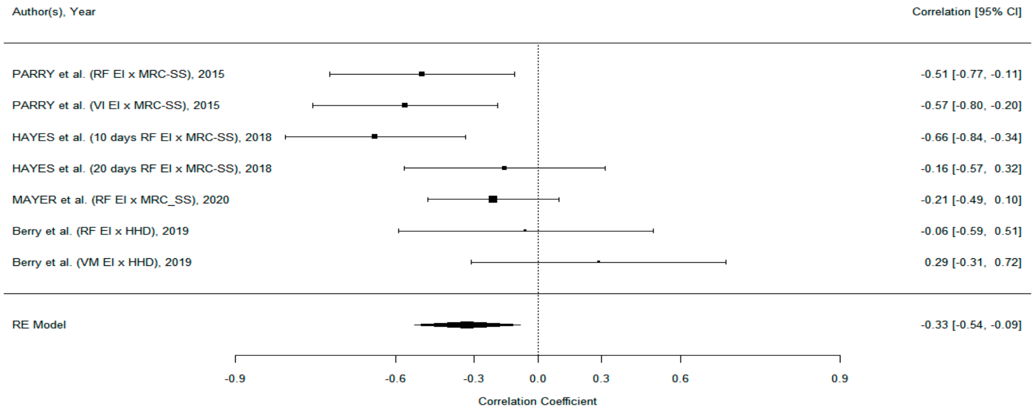


EI: Echo intensity; RF: Rectus Femoris; MRC-SS: Medical Research Council Sum Score; HHD: Hand Held Dynamomenter;

Figure S22: Forest plot of the sensitivity analysis of the correlation between EI and mobility

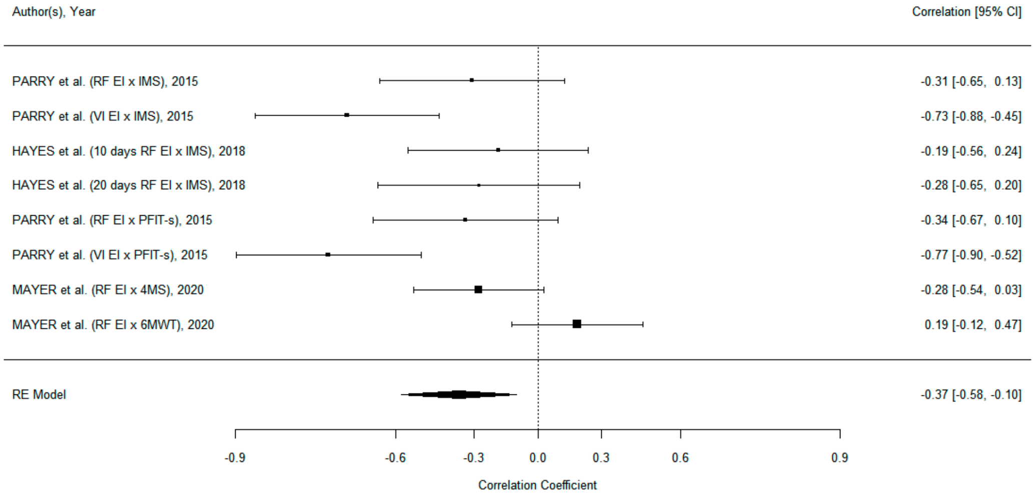


EI: Echo intensity; RF: Rectus Femoris; VI: vastus intermedius; IMS: ICU mobility scale; PFIT-s: Physical Function in Intensive Care Test; 5 x STS: Five Times Sit to Stand Test; 4MGS: 4 meter gait speed test; 6MWT: 6-minute walk test.

Figure S23: Funnel plot of the correlation between MT and muscle strength

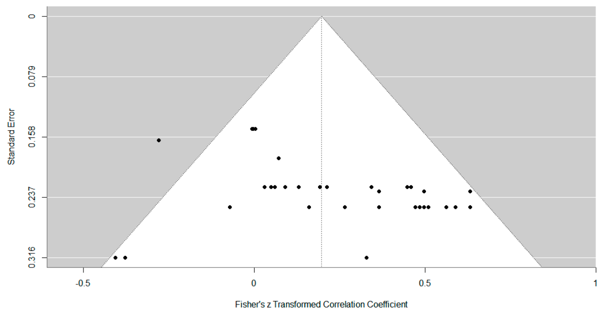


Figure S24: Funnel plot of the correlation between MT of rectus femoris and muscle strength assessed by MRC-SS

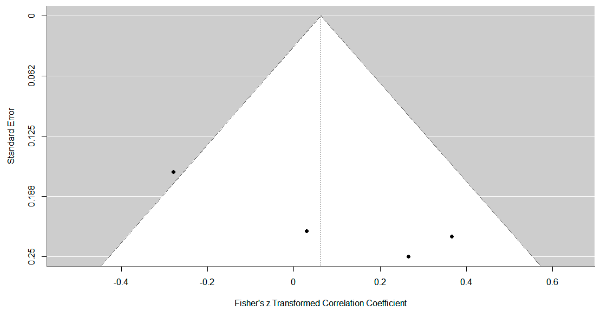


Figure S25: Funnel plot of the correlation between MT of vastus intermedius and muscle strength assessed by MRC-SS

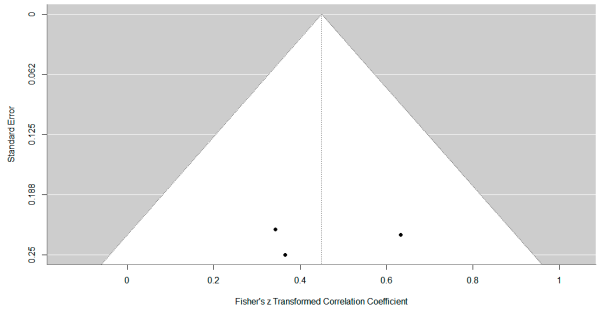

Figure S26: Funnel plot of the correlation between MT of vastus lateralis and muscle strength assessed by MRC-SS

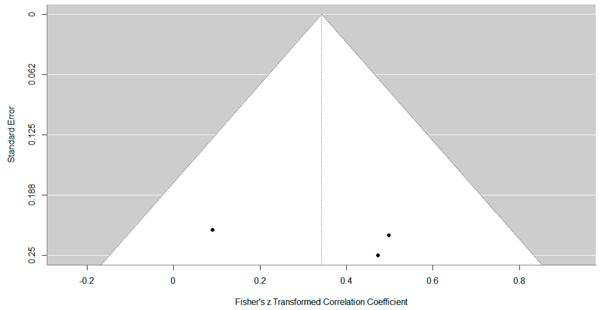


Figure S27: Funnel plot of the correlation between MT of rectus femoris and muscle strength assessed by HHD

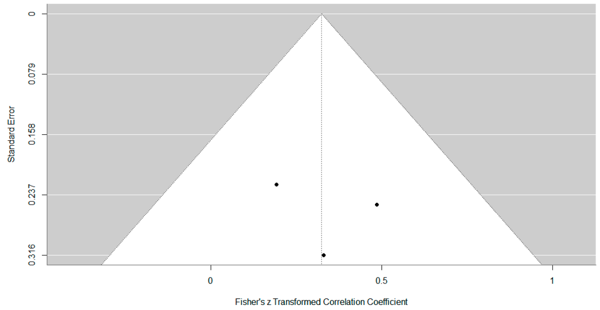


Figure S28: Funnel plot of the correlation between MT of vastus lateralis and muscle strength assessed by HHD

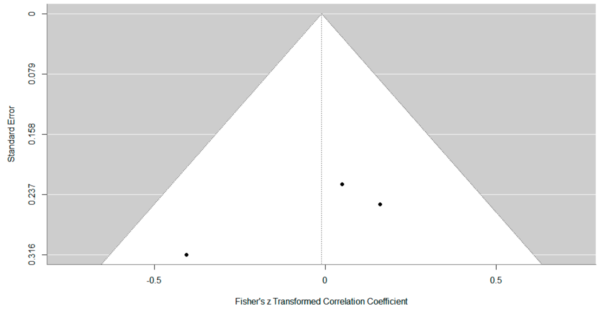

Figure S29: Funnel plot of the correlation between CSA and muscle strength

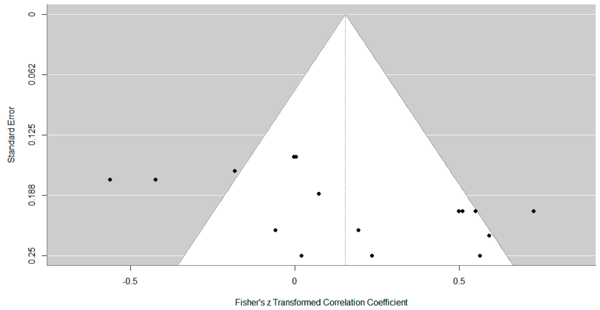


Figure S30: Funnel plot of the correlation between CSA of rectus femoris and muscle strength assessed by MRC-SS

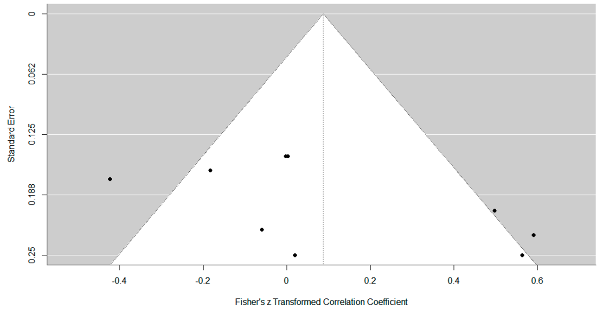


Figure S31: Funnel plot of the correlation between CSA of rectus femoris and muscle strength assessed by HGS

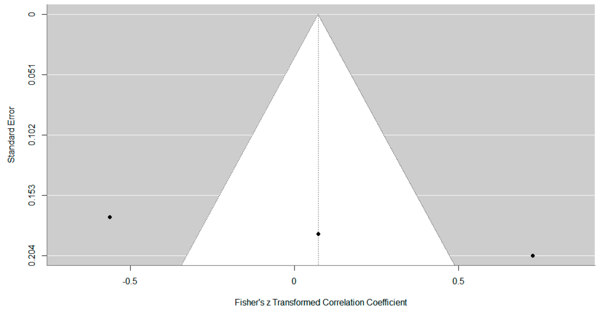

Figure S32: Funnel plot of the correlation between EI and muscle strength

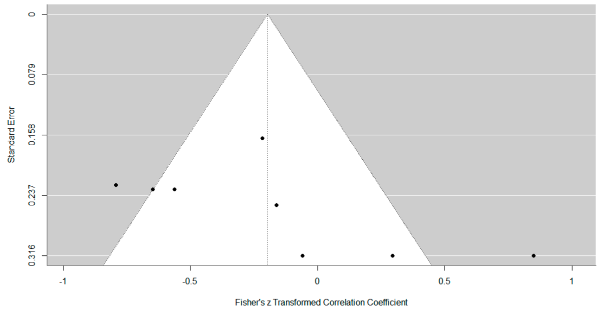


Figure S33: Funnel plot of the correlation between EI of rectus femoris and muscle strength

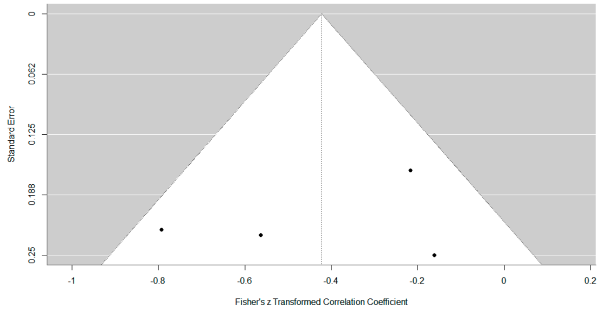


Figure S34: Funnel plot of the correlation between MT and mobility

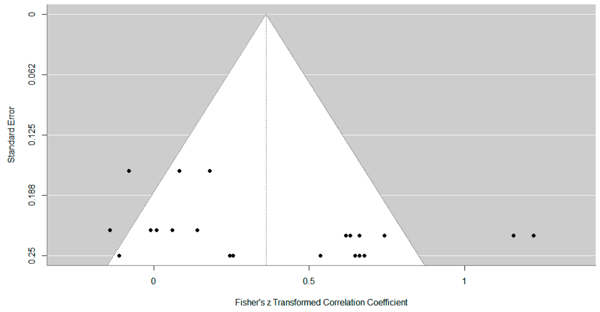

Figure S35: Funnel plot of the correlation between MT of rectus femoris and mobility

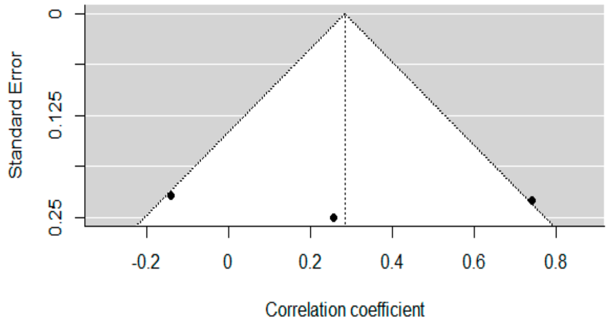


Figure S36: Funnel plot of the correlation between MT of vastus intermedius and mobility

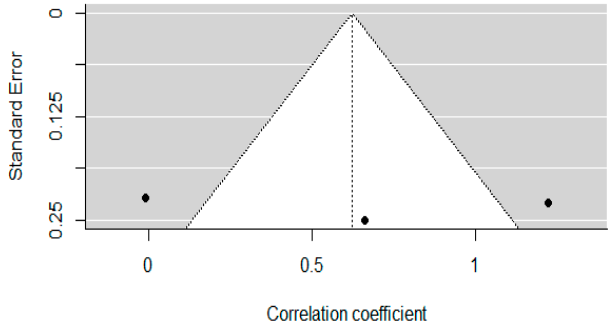


Figure S37: Funnel plot of the correlation between MT of vastus lateralis and mobility

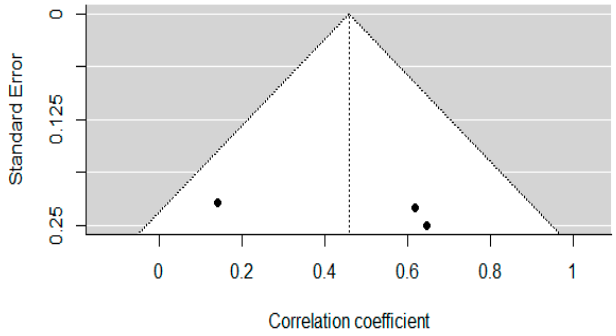

Figure S38: Funnel plot of the correlation between CSA and mobility

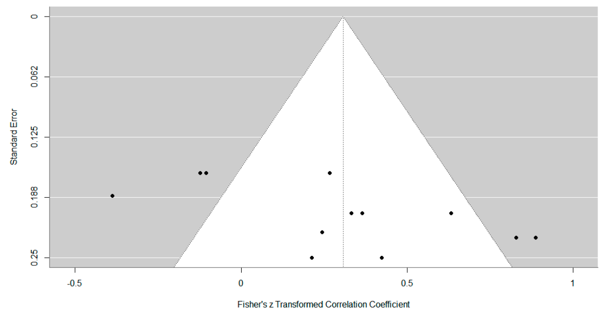


Figure S39: Funnel plot of the correlation between CSA of rectus femoris and mobility

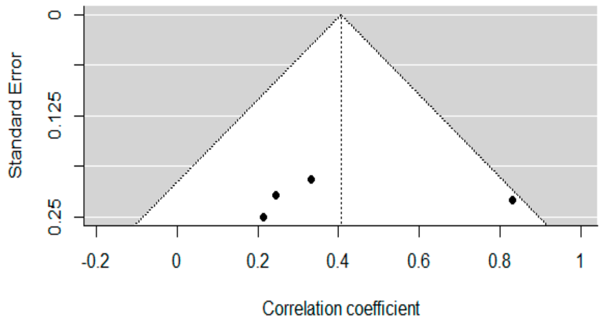


Figure S40: Funnel plot of the correlation between EI and mobility

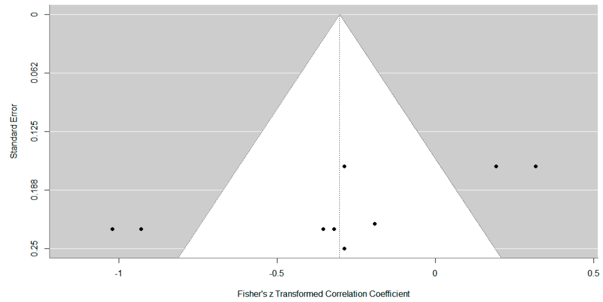

Figure S41: Funnel plot of the correlation between EI of rectus femoris and mobility

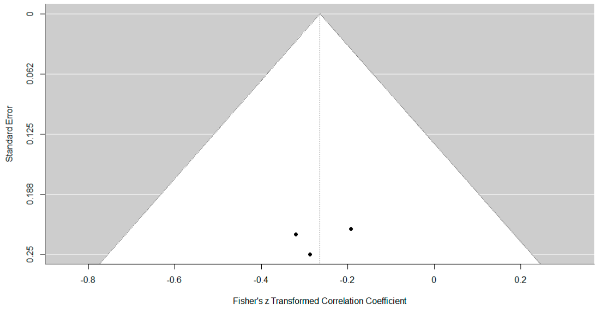


Figure S42: Funnel plot of the sensitivity analysis of the correlation between EI and muscle strength

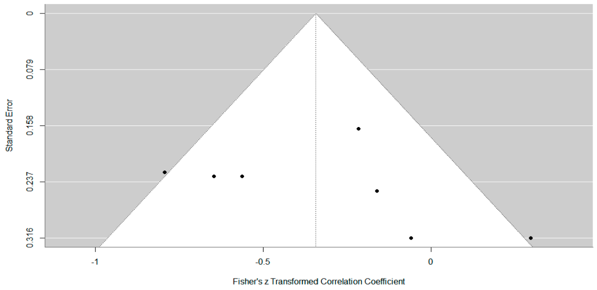


Figure S43: Funnel plot of the sensitivity analysis of the correlation between EI and mobility

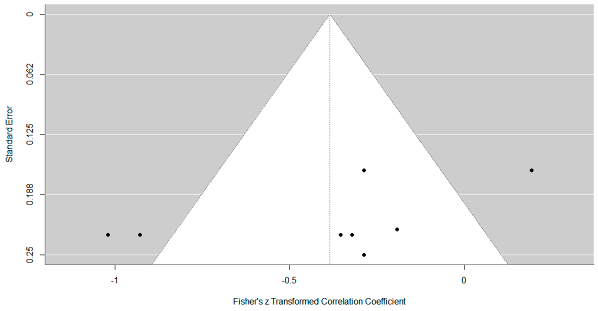

Supplement: Supplementary file 1 [file ijerph-21-00908-s001.zip › ijerph-3084205-supplementary.pdf]
